# HIV/AIDS treatment failure and associated factors in Ethiopia: meta-analysis

**DOI:** 10.1186/s12889-020-8160-8

**Published:** 2020-01-20

**Authors:** Aklilu Endalamaw, Mengistu Mekonnen, Demeke Geremew, Fikadu Ambaw Yehualashet, Hiwot Tesera, Tesfa Dejenie Habtewold

**Affiliations:** 1 Department of Pediatrics and Child Health Nursing, School of Health Sciences, College of Medicine and Health Sciences, Bahir Dar University, P.O. Box 196, Bahir Dar, Ethiopia; 2 Department of Pediatrics and Child Health Nursing, School of Nursing, College of Medicine and Health Sciences, University of Gondar, Gondar, Ethiopia; 3 Department of Immunology, School of Biomedical and Laboratory Sciences, College of Medicine and Health Sciences, University of Gondar, Gondar, Ethiopia; 4 Department of comprehensive nursing, School of Nursing, College of Medicine and Health Sciences, University of Gondar, Gondar, Ethiopia; 5 Student Clinic, Microbiologist, Bahirdar University, Bahir Dar, Ethiopia; 6 Department of Epidemiology, University of Groningen, Groningen, The Netherlands

**Keywords:** HAART, HIV, Failure, Treatment, Ethiopia

## Abstract

**Background:**

The national burden of human immunodeficiency virus treatment failure and associated factors in the Ethiopian context is required to provide evidence towards a renewed ambitious future goal.

**Methods:**

We accessed Ethiopian Universities’ online repository library, Google Scholar, PubMed, Web of Science, and Scopus to get the research articles. We run I-squared statistics to see heterogeneity. Publication bias was checked by using Egger’s regression test. The pooled prevalence was estimated using the DerSimonian-Laird random-effects model. We employed the sensitivity analysis to see the presence of outlier result in the included studies.

**Results:**

The overall human immunodeficiency treatment failure was 15.9% (95% confidence interval: 11.6–20.1%). Using immunological, virological, and clinical definition, human immunodeficiency treatment failure was 10.2% (95% confidence interval: 6.9–13.6%), 5.6% (95% confidence interval: 2.9–8.3%), and 6.3% (95% confidence interval: 4.6–8.0%), respectively. The pooled effects of World Health Organization clinical stage III/IV (Adjusted Odd Ratio = 1.9; 95% CI: 1.3–2.6), presence of opportunistic infections (Adjusted Odd Ratio = 1.8; 95% CI: 1.2–2.4), and poor adherence to highly active antiretroviral therapy (Adjusted Odd Ratio = 8.1; 95% CI: 4.3–11.8) on HIV treatment failure were estimated.

**Conclusions:**

Human immunodeficiency virus treatment failure in Ethiopia found to be high. Being on advanced clinical stage, presence of opportunistic infections, and poor adherence to highly active antiretroviral therapy were the contributing factors of human immunodeficiency virus treatment failure. Human immunodeficiency virus intervention programs need to address the specified contributing factors of human immunodeficiency virus treatment failure. Behavioral intervention to prevent treatment interruption is required to sustain human immunodeficiency virus treatment adherence.

**Protocol registration:**

It has been registered in the PROSPERO database with a registration number of CRD42018100254.

## Background

Globally, there were approximately 37.9 million Human Immunodeficiency Virus (HIV) infected people and around 770,000 people died from AIDS-related illnesses worldwide in 2018. In this year, there were 20.6 million people with HIV in eastern and southern Africa, and 5.0 million in western and central Africa [1]. In Ethiopia, 690,000 people were living with HIV in 2018 [2].

In 2018, 23.3 million people with HIV were accessing antiretroviral therapy (ART) worldwide [1]. In the same year, 65% of people living with HIV were on treatment in Ethiopia [2]. A review of the HIV situation in Addis Ababa Ethiopia revealed that weak monitoring of the quality of interventions, limited linkage of HIV-positive clients, lost to follow-up, financial shortage, limited man-power, and gaps in the use of program data were the challenges of HIV/AIDS treatment [3].

The risk of death due to HIV has been decreased after the era of highly active antiretroviral therapy (HAART) [4]. Evidence has shown that an individual on HAART with an undetectable viral load, absence of an advanced clinical finding, and high CD4 count are less likely to transmit HIV to another person [5, 6]. However, the risk of HIV transmission is high due to treatment failure. Treatment failure can be a virological, immunological, or clinical failure [7]. Virological failure is a plasma viral load above 1000 copies/ ml based on two consecutive viral load measurements after 3 months with adherence support [7]. Immunological failure is when the CD4 count falls to the baseline (or below) or persistent CD4 levels below 100 cells/mm3 for adult and adolescent or below 200 cells/mm3 in younger than 5 years. Clinical failure is defined as the occurrence or recurrence of advanced WHO clinical stage after 6 months of therapy [7].

Globally, UNAIDS planned to have 90% of people on HAART are virally suppressed by 2030 and as a result, HIV treatment failure would be prevented [8]. Despite this ambitious goal, as of a systematic analysis of national HIV treatment cascades of 69 countries by 2016, viral suppression was between 7% in China and 68% in Switzerland [9]. It can be prevented through the implementation of globally recommended strategies. For instance, improving HAART adherence, taking medication based on the appropriate prescription, prevent drug-drug interaction, increasing knowledge and attitudes of patients towards HAART, timely initiation of HAART, prevention and control of opportunistic infections, and implementation of effective food and nutrition policy.

A higher viral load may lead to HIV treatment failure, which is becoming a threat of different African countries, like in Burkina Faso (6.4%) [10], Ghana (15.7%) [11], and Tanzania (14.9%) [12]. In Ethiopia, virological, immunological, and clinical failure is found in the range between 1.3% [13] to 11.5% [14], 2.1% [15] to 21% [16], and 3.1% [17] to 12.3% [18], respectively.

With these variations of reports, there is no pooled representative national data in Ethiopia. In order to provide evidence towards a renewed ambitious future goal, it is now critical to reflect the pooled burden of HIV treatment failure in the Ethiopian context. The objective of this study was first, to estimate the national burden of HIV treatment failure and secondly, to review contextual factors of HIV treatment failure using globally accepted key performance indicators as a framework. Thus, this information will be helpful for healthcare professionals and further helps to enable the country to sustain successes and improve weaknesses towards the goal of ending AIDS strategy.

## Methods

### Reporting

It is reported based on the Preferred Reporting Items for Systematic Reviews and Meta-analyses (PRISMA) guideline [19] (supplementary file-research checklist). Its protocol is registered in the Prospero database with a registration number of CRD42018100254.

### Search strategy

PubMed, Web of Science, Scopus, and Google Scholar databases were used to get the research articles. The search strategy made in PubMed was: [(“Human Immunodeficiency virus”[MeSH Terms] OR HIV OR AIDS OR “Acquired Immunodeficiency syndrome” AND (“antiretroviral therapy”[MeSH Terms] OR “highly antiretroviral therapy” OR HAART OR ART OR “ARV Therapy” OR “antiretroviral therapy”) AND (outcome OR “treatment failure” OR failure OR “virological failure” OR “immunological failure” OR “Clinical failure”) AND (Ethiopia)]. The search done in PubMed through search terms was 03/10/2018. In addition, Ethiopian Universities’ (University of Gondar and Addis Ababa University) online repository library were searched. Endnote 7 reference manager software was used to manage duplicated references and for citation in the text.

### Inclusion and exclusion criteria

Those articles included in this meta-analysis were: [1] cohort, case-control, and cross-sectional studies, [2] studies that reported the prevalence and/ or AOR (adjusted odds ratio) of associated factors of overall HAART treatment, immunological, clinical, and virological failure, [3] studies conducted in Ethiopia, and [4] studies published in English.

Studies without full-text access, qualitative studies, and conference proceeding without full-text reports were excluded.

### Outcome measurement

According to WHO [7], HIV treatment failure could be a clinical, immunological, and virological failure.

The prevalence of failure was ascertained by dividing the participants with the outcome of interests to the overall study participants multiplied by 100.

### Quality assessment

Two authors assessed the quality of the articles based on the Newcastle-Ottawa Scale quality assessment tool for cross-sectional, case-control, and cohort studies [20]. The criteria for cross-sectional studies have three sections, in which the first section focused on selection and graded by four stars, the second section dedicated with the comparability of the study and graded by two stars, and the third section emphasized on the outcome and graded by three stars. The criteria for case-control studies were: 1) selection evaluated by four stars, 2) comparability assessed by two stars, and 3) exposure graded by four stars. The criteria for cohort studies were: 1) selection graded by six stars, 2) comparability graded by two stars, and 3) outcome graded by five stars. Whenever disagreement happened between the two quality assessors, the procedure would be repeated and further solved with the involvement of the third reviewer. Cross-sectional, case-control, and cohort studies scored 6 and/or above, 7 and/or above, and 9 and/or above quality assessment criteria were included respectively.

### Data extraction process

Two authors extracted the required data. The first author and year of publication, sample size, an outcome of interest, study design, study population, the geographical location of the study, fund, and response rate were collected.

### Data synthesis and statistical analysis

STATA 14 (Stata Corp, College Station, TX, USA) statistical software was used for meta-analysis. Publication bias assessed by the funnel plot and more objectively by Egger’s regression test. I-squared statistics was used to check the heterogeneity of the studies. The DerSimonian-Laird random-effects model was employed to estimate the overall prevalence. Subgroup analysis based on the geographical location of the study, type of treatment failure, study population by age, and study design was conducted to see the variation in outcomes. The sensitivity analysis was also employed to see whether the outlier result found in the included studies.

## Results

### Search results

**Fig. 1:**
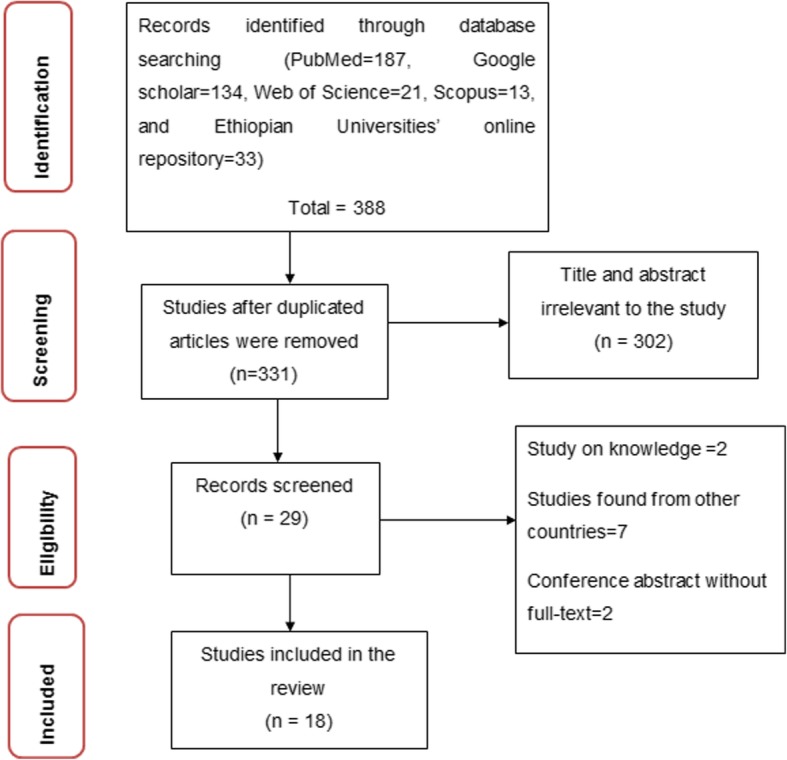
PRISMA flow-chart diagram describing the selection of studies

A total of 873 articles were found from PubMed (*n* = 187), Google Scholar (*n* = 134), Web of Science (*n* = 21), Scopus (n = 13), and Ethiopian Universities’ online repository library (University of Gondar and Addis Ababa University) (*n* = 33). A total of 331 articles have remained after duplicate studies were removed. Then, 302 articles were removed based on the unmatched title and abstracts. Finally, 18 articles were included (Fig. 1).

### Characteristics of studies

Studies found through databases search were done between 2005 and 2016. Eight of the studies were conducted in the Amhara region, whereas five in Addis Ababa [13, 21–24], three in Oromia [18, 25, 26], one in Tigray [14], and one in SNNPR [27]. Three studies were done by case-control study design [24, 28, 29], four studies by cross-sectional [14, 30–32], and eleven by cohort study design [13, 15–18, 21–23, 25–27]. Ten studies were done on adult population [13, 16, 17, 21, 23–25, 28, 29, 32], six on children [15, 18, 22, 26, 27, 31], and two on all age group [14, 30] (Table 1).

**Table 1:** Characteristic of included studies in systematic review and meta-analysis

First Author/Year	Study period	Region	Study design	Study population	Sample size	Response rate	Source of fund
Teshome W/2015 [28]	2007–2009	Addis Ababa	Retrospective cohort	Adult	293	100%	Not reported
Bokretsion BG et al./2017 [23]	2016	Amhara	Cross-sectional	All age group	421	100%	Bahirdar University and Ethiopian public health institute
Yassin S/2017 [20]	2006–2015	Oromia	Retrospective cohort	children	269	86.8%	Not reported
Zeleke A/2016 [24]	2005–2013	Amhara	Retrospective cohort	children	225	100%	Not reported
Yimer YT/2015 [15]	2009–2013	Addis Ababa	Retrospective cohort	Adult	525	100%	Not reported
Bacha T et al./2012 [29]	2005–2011	Addis Ababa	Retrospective cohort	children	1186	100%	Not reported
Ayalew MB et al./2016 [25]	2011–2015	Amhara	Retrospective study	Adult	340	100%	University of Gondar, Ethiopia
Sisay MM et al./2018 [17]	2010–2016	Amhara	Retrospective cohort	children	824	81.9%	University of Gondar, Ethiopia
Tsegaye AT et al./2016 [19]	2006–2014	Amhara	Retrospective cohort	Adult	356	100%	University of Gondar,Ethiopia
Hailu GG et al./2017 [16]	2008–2016	Tigray	Cross-sectional	All age group	260	100%	Mekelle Univesity, Ethiopia
Yayehirad AM et al./2013 [18]	2007–2008	Amhara	Retrospective cohort	Adult	509	100%	University of Gondar,Ethiopia
Abdissa A et al./2014 [32]	2010–2012	Oromia	Prospective cohort	Adult	265	100%	Danish International Development Agency (DANIDA)
Tadesse BT et al. /2017 [33]	2015–2016	SNNPR	cohort	children	628	100%	Hawassa University, Ethiopia
Workneh N et al./2009 [34]	2005–2008	Oromia	Retrospective cohort	children	96	100%	Jimma University, Ethiopia
Sisay C et al./2017 [30]	2011–2016	Addis Ababa	Retrospective cohort	Adult	595	100%	Ethiopian public health institute
Babo YD et al./2017 [26]	2014	Amhara	Case-control	Adult	304	100%	USAID
Bayu B et al./2017 [27]	2015	Amhara	Case-control	Adult	306	100%	Not reported
Getnet Y /2014 [31]	2005–2011	Addis Ababa	Case-control	Adult	309	100%	Jimma University, Ethiopia

### Publication bias

**Fig. 2:**
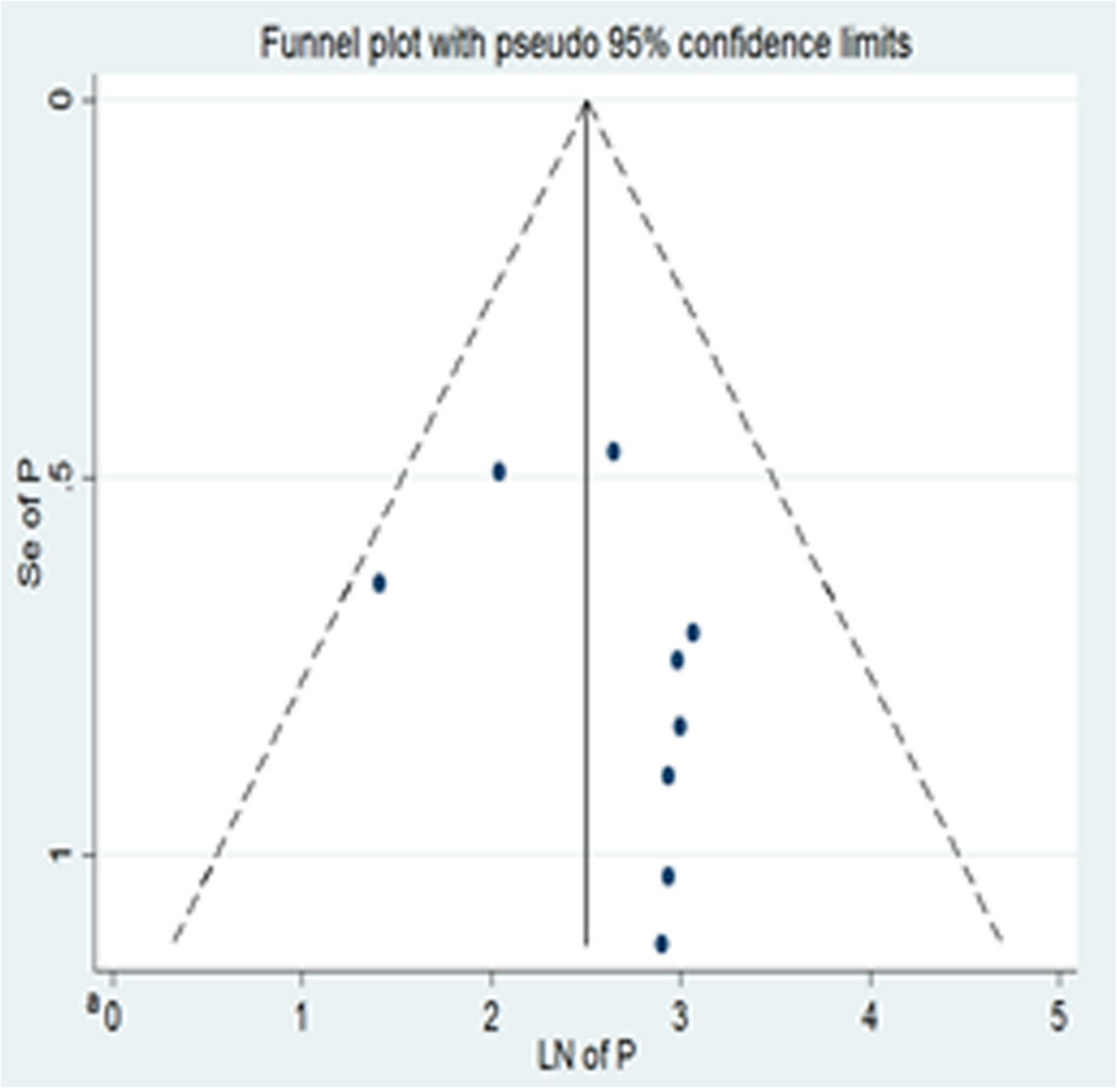
Funnel plot, in which the vertical line indicates the effect size whereas the diagonal line indicates the precision of individual studies with 95% confidence limit

The funnel plot for HIV treatment failure is shown below (Fig. 2). Egger’s regression test of the *p*-value for overall HIV treatment failure is 0.226.

### Meta-analysis

#### HIV treatment failure based on the definition of HAART failure

**Fig. 3:**
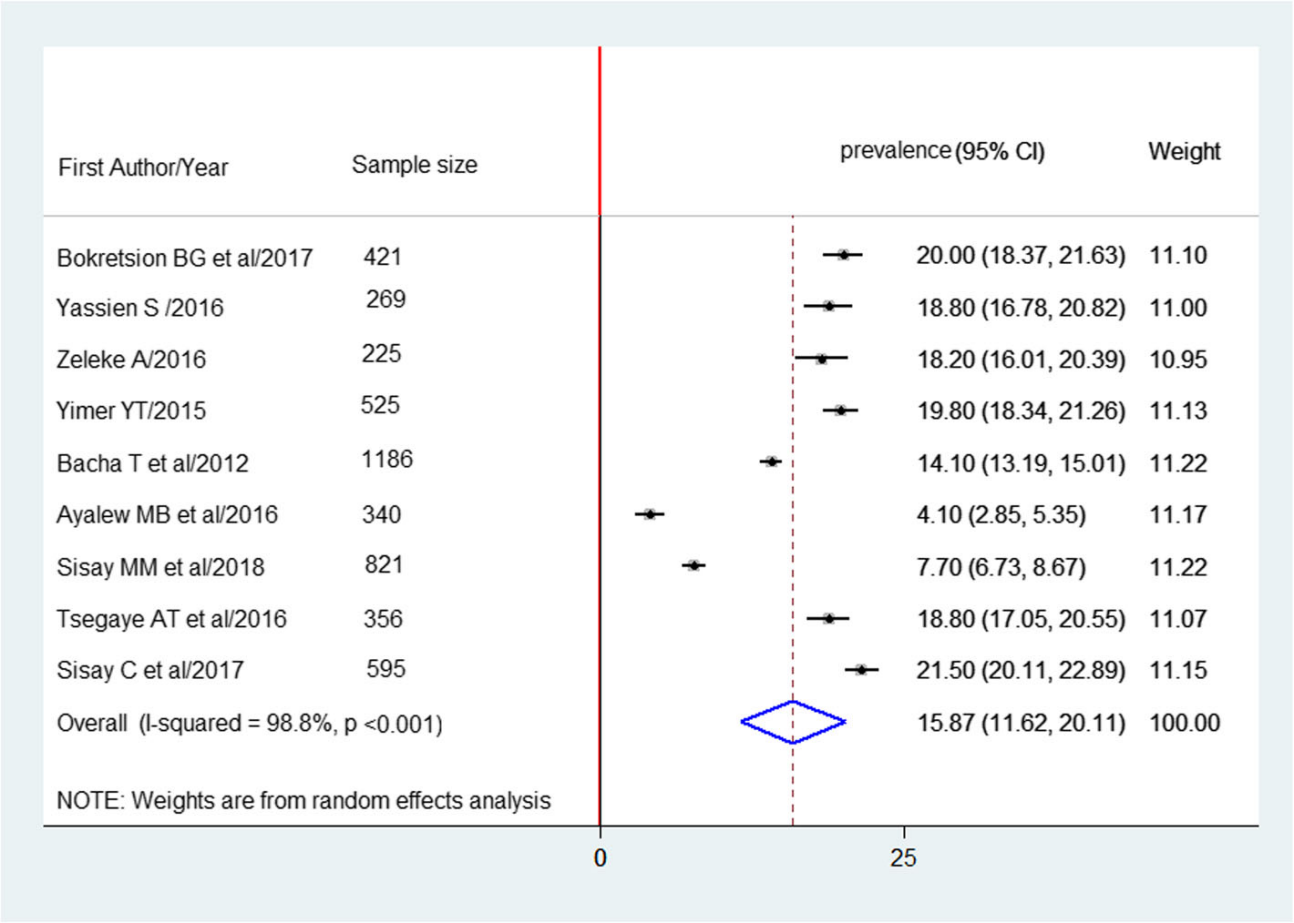
Forest plot of the prevalence of HAART failure in Ethiopia and its 95%CI, the midpoint of each line illustrates the prevalence rate estimated in each study. The diamond shows pooled prevalence

A total of 4738 participants in nine studies were used to estimate the pooled prevalence of HIV treatment failure based on the definition of HAART failure. The pooled prevalence of HIV treatment failure was 15.9% (95% CI: 11.6–20.1%) (Fig. 3).

#### Immunological and Virological definition of HIV treatment failure

**Fig. 4:**
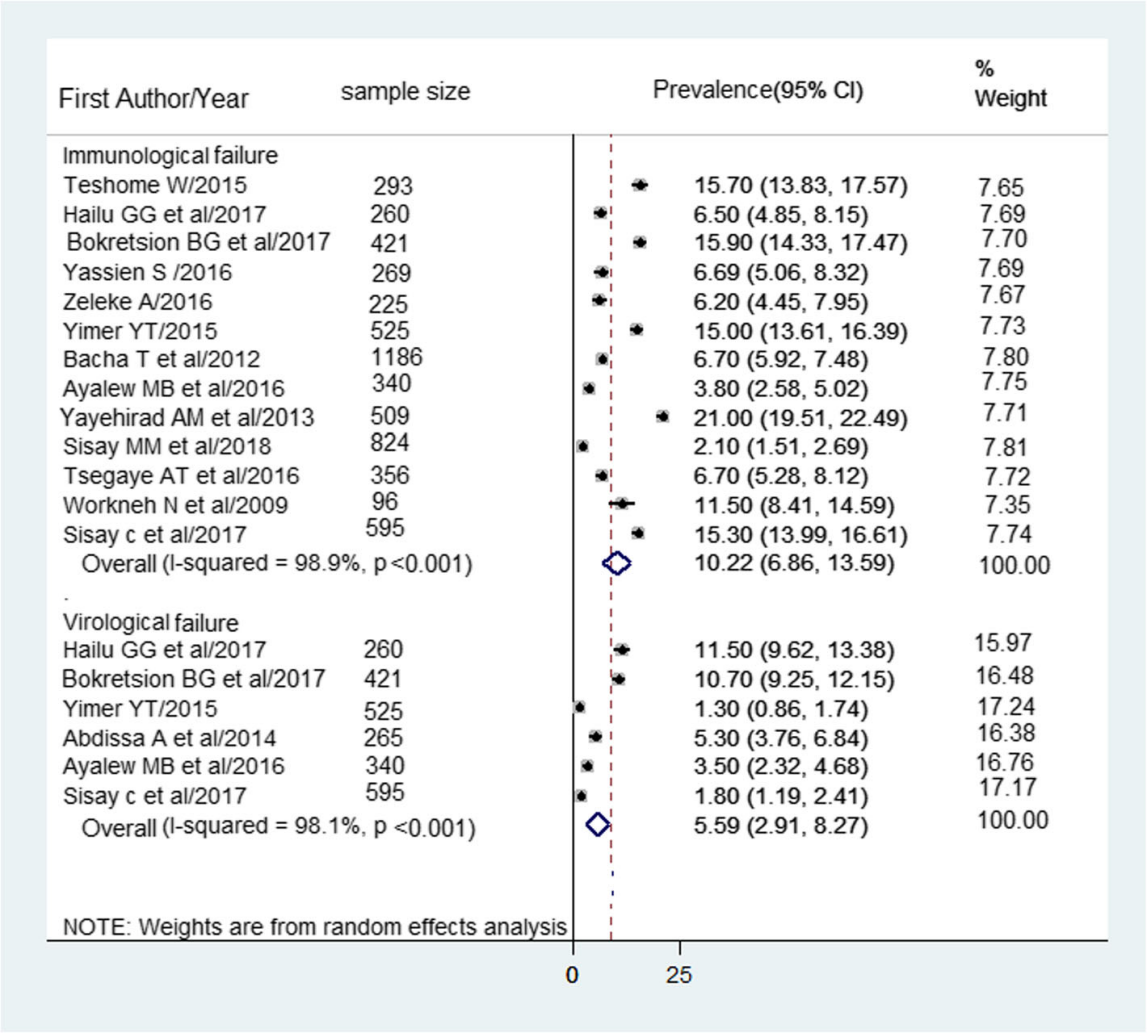
Forest plot of the prevalence of immunological and virological failure in Ethiopia and its 95%CI, the midpoint of each line illustrates the prevalence rate estimated in each study. The diamond shows pooled prevalence

A total of 5899 study participants in 13 studies were involved to determine HIV treatment failure based on the immunological definition. Of which, 10.2% (95% CI: 6.9–13.6%) developed immunological failure. Regarding virological failure, the pooled prevalence from six studies with a total of 2406 participants was 5.6% (95% CI: 2.9–8.3%) (Fig. 4).

#### Clinical definition of HIV treatment failure

**Fig. 5:**
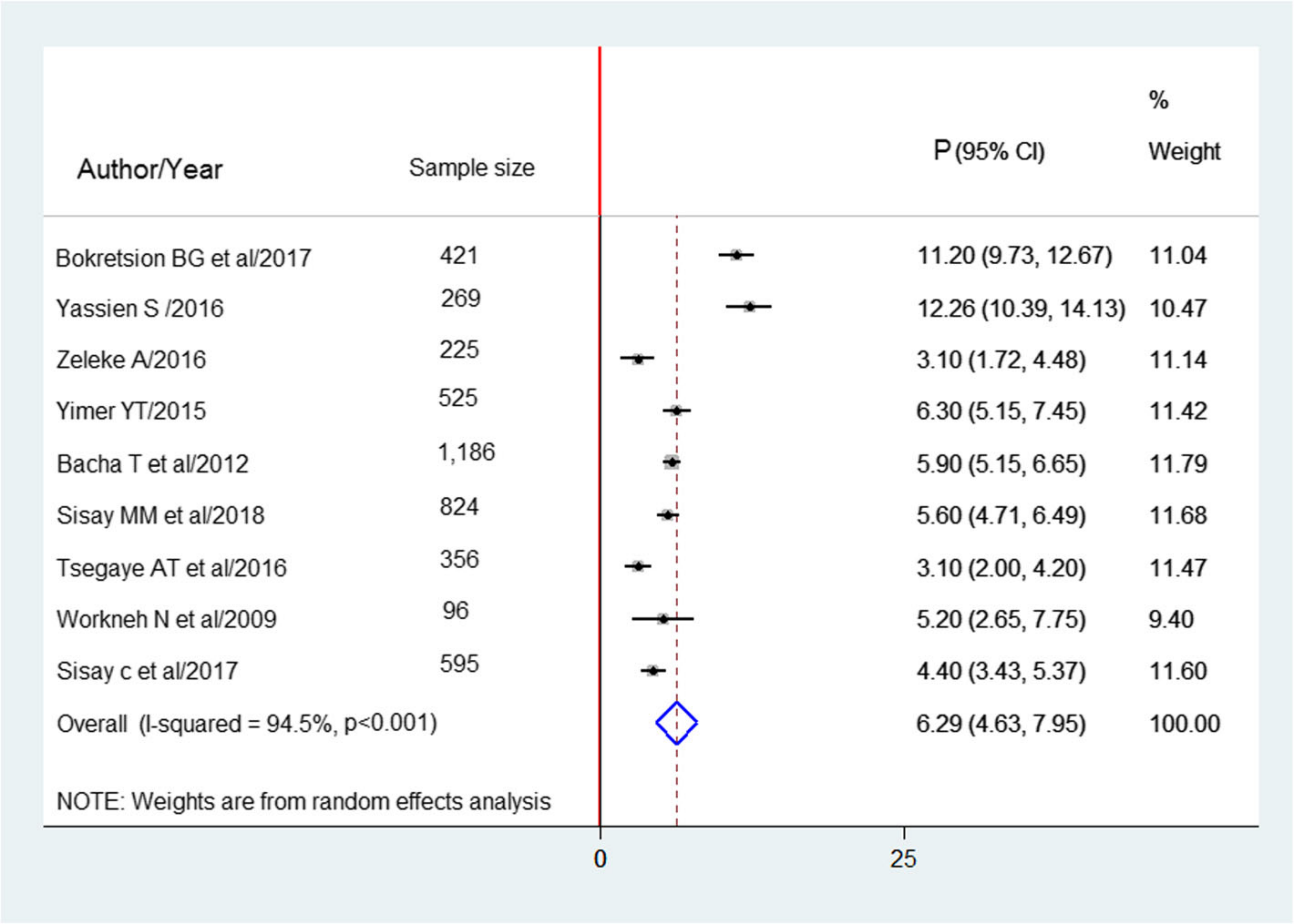
Forest plot of the prevalence of clinical failure in Ethiopia and its 95%CI, the midpoint of each line illustrates the prevalence rate estimated in each study. The diamond shows pooled prevalence

A total of 4497 study participants in 9 studies were found to estimate the clinical failure, in which the pooled prevalence was 6.3% (95% CI: 4.6–8.0%) (Fig. 5).

### Subgroup analysis

Subgroup analysis was employed based on region, age of the study participants, and study design. Lower prevalence of HIV treatment failure based on the definition of HAART, immunological, and virological failure was 13.7%in Amhara, 6.5% in Tigray, and 1.5% in Addis Ababa, respectively (Table 2).

**Table 2:** Subgroup analysis of the prevalence (p) of HIV treatment failure based on overall HAART, immunological, virological, and clinical definition by region, age, and study design in Ethiopia

Subgroup analysis	Overall HAART failure P (95%CI)	Immunological failure P (95%CI)	Virological failure P (95%CI)	Clinical failure P (95% CI)
By Region				
Amhara	13.7 (7.3–20.2)	9.3 (3.3–15.2)	7.1 (0.03–14.1)	5.7 (2.6–8.9)
Oromia	18.8 (16.8–20.8)	8.9 (4.2–13.6)	5.3 (3.8–6.8)	8.8 (1.9–15.7)
Addis Ababa	18.4 (13.6–23.3)	13.2 (7.9–18.4)	1.5 (1.0–2.0)	5.5 (4.4–6.6)
Tigray	__	6.5 (4.9–8.2)	11.5 (9.6–13.4)	__
By age of participants				
All age group	20.0 (18.4–21.6)	11.2 (2.0–20.4)	11.0 (9.9–12.2)	11.2 (9.7–12.7)
Adult	16.0 (7.4–24.7)	12.9 (7.6–18.3)	2.8 (1.5–4.1)	4.6 (2.9–6.3)
Children	14.6 (9.7–19.6)	6.4 (3.6–9.3)	__	6.4 (4.2–8.5)
By Study design				
Cross-sectional	14.1 (3.0–25.2)	8.1 (2.7–13.5)	8.5 (3.2–13.9)	7.2 (0.8–15.8)
Cohort	16.8 (12.2–21.37)	11.2 (6.8–15.6)	2.6 (1.1–4.0)	6.0 (4.5–7.5)
Combined	15.8 (11.6–20.1)	10.2 (6.9–13.6)	5.6 (2.9–8.3)	6.3 (4.6–8.0)

*__ denotes no estimation due to lack of original studies*

### Sensitivity analysis

In the sensitivity analysis, the overall HIV treatment failure based on the definition of HAART failure was observed high (17.3%) and low (15.2%) when *Ayalew MB* et al *2016* and *Sisay C* et al*/2017* was omitted respectively. The minimum pooled prevalence of HIV treatment failure based on immunological definition (9.3%), virological definition (4.4%), and clinical definition (5.5%) when *Yayehirad AM* et al*/2013, Hailu GG* et al */2015, and Yassin S /2016* omitted, respectively. And the maximum pooled prevalence of HIV treatment failure based on immunological definition (10.8%) and virological failure (6.5%) *Ayalew MB* et al*/2016* and *Yimer YT/2015* was dropped from the analysis, respectively (Table 3).

**Table 3:** The prevalence (p) of HIV treatment failure based on HAART failure, immunological, virological, and clinical definition when the study omitted in Ethiopia

Study omitted	HAART failure P (95%CI)	Immunological failure P (95%CI)	Virological failure P (95%CI	Clinical failure P (95% CI)
Bokretsion BG et al./2017	15.3 (10.8–19.9)	9.7 (6.3–13.2)	4.5 (2.3–6.7)	5.6 (4.2–7.1)
Yassin S /2017	15.5 (10.9–20.1)	10.5 (6.9–14.1)	__	5.5 (4.1–7.1)
Zeleke A/2016	15.6 (11.0–20.2)	10.6 (7.0–14.1)	__	6.7 (4.9–8.4)
Yimer YT/2015	15.4 (10.8–19.9)	9.8 (6.4–13.3)	6.5 (2.7–10.3)	6.3 (4.4–8.2)
Bacha T et al./2012	16.1 (10.9–21.3)	10.5 (6.6–14.4)	__	6.4 (4.3–8.4)
Ayalew MB et al./2016	17.3 (13.5–21.2)	10.8 (7.1–14.4)	6.0 (2.9–9.2)	__
Sisay MM et al./2018	16.9 (12.6–21.2)	10.9 (7.7–14.1)	__	6.4 (4.4–8.4)
Tsegaye AT et al./2016	15.5 (10.9–20.1)	10.5 (6.9–14.1)	__	6.7 (5.0–8.4)
Teshome W/2015	__	9.8 (6.3–13.2)	__	__
Hailu GG et al./2015	__	10.5 (6.9–14.1)	4.4 (2.0–6.9)	__
Yayehirad AM et al./2013	__	9.3 (6.3–12.4)	__	__
Workneh N et al./2009	__	10.1 (6.6–13.6)	___	6.4 (4.6–8.2)
Sisay C et al./2017	15.2 (10.8–19.5)	9.8 (6.4–13.2)	6.4 (2.4–10.4)	6.5 (4.7–8.4)
Abdissa A et al./2014	__	__	5.6 (2.7–8.6)	__
Combined	15.8 (11.6–20.1)	10.2 (6.8–13.6)	5.6 (2.9–8.3)	6.3 (4.6–7.9)

*__ denotes no estimation due to lack of original studies*

### Associated factors of HIV treatment failure

HIV treatment failure is attributed to socio-demographic, clinical, drug, and health system-related factors.

#### Socio-demographic factors

Based on a single study report, children’s age between 6 and 9 years (AOR = 0.26; 95% CI: 0.09–0.72) was protective towards HIV treatment failure as compared to 10–15 years old children [18]. Another study showed children less than 3 years old were high risk (AHR = 1.85; 95% CI: 1.24–2.76) for HIV treatment failure as compared to 5–15 years old children [22].

One study which was done on the adult population [29] showed that those aged < 35 years were high risk (AOR = 2.5; 95% CI: 1.3–4.8) to develop HIV treatment failure as compared to their counterparts.

From a single study, male adult patients (AOR = 4.6; 95% CI: 1.7–12.3) [14], and patients in the formal educational level (AOR = 5.15; 95% CI: 1.5–17.3) [28] were at higher risk for HIV treatment failure.

*Babo YD* et al*/2017* (AOR = 4.9; 95% CI: 1.5–16) and *Yayehirad MA* et al*/2013* (AOR = 1.7; 95% CI: 1.1–2.7) [16, 28] found that the odds of being unemployed is more likely to develop HIV treatment failure.

#### Clinical-related factors

Report from one study showed that lower baseline body mass index (BMI) (AOR = 2.8; 95% CI: 1.01–7.5) [28] and patients who had height for age in the third percentile or less (AHR = 3.3; 95% CI: 1.0–10.6) [22] were more likely to expose to HIV treatment failure. On the other hand, weight change per 1 kg increase (AHR = 0.9, 95% CI: 0.9–0.9) [17], and < 50 kg weight at baseline (AHR = 0.58, 95% CI:0.38–0.89) [13] were less likely to expose to HIV treatment failure.

One study showed [16], being in ambulatory functional status was at high risk (AOR = 2.9, 95%CI: 1.2–7.5) to develop HIV treatment failure than being in working functional status.

Another study [15] showed that those children who did not know their HIV status were at high risk (AHR = 4.4, 95% CI: 1.8–11.3) to develop HIV treatment failure.

**Fig. 6:**
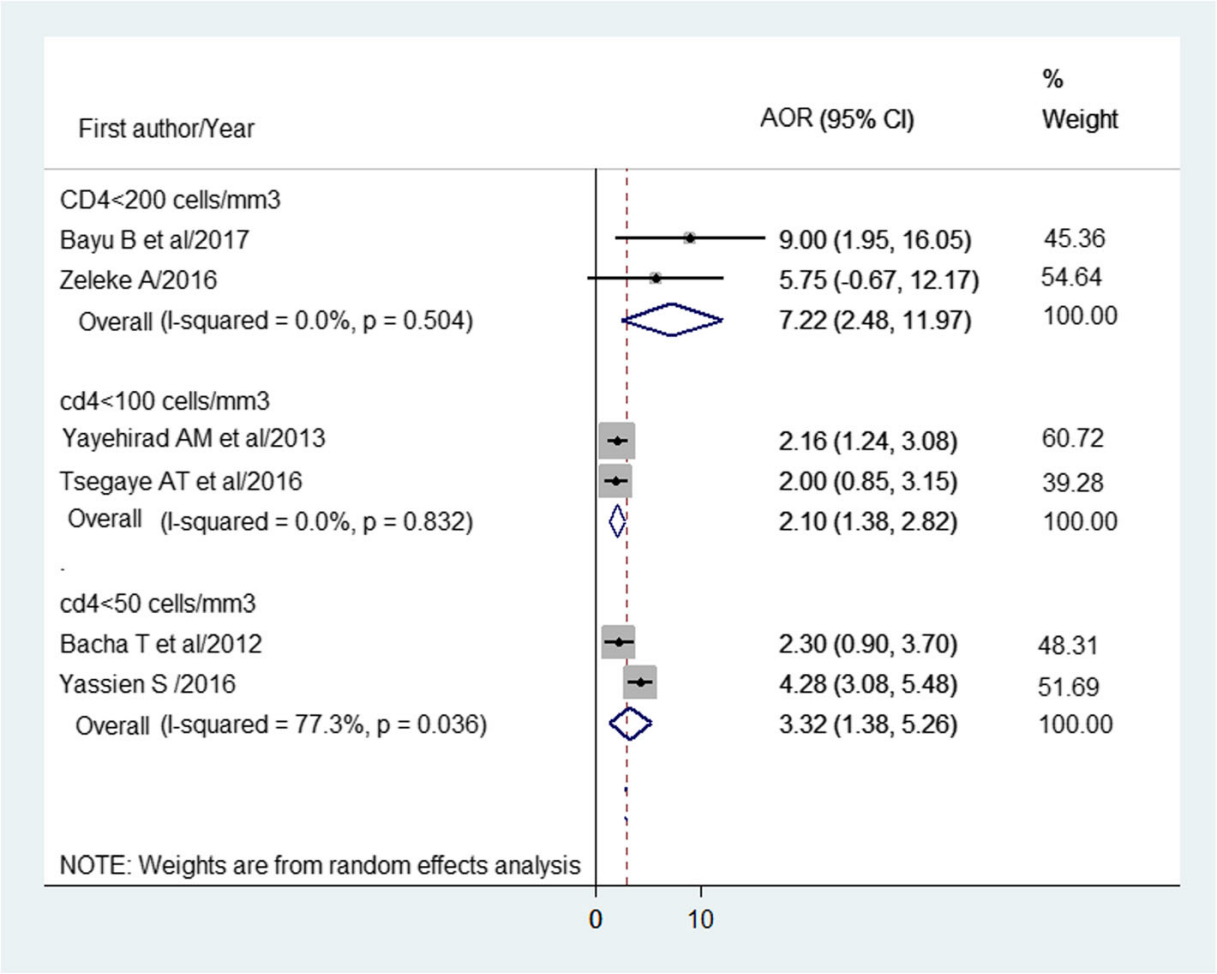
Forest plot of the adjusted odds ratios (AOR) with corresponding 95% CI of studies on the association of CD4 cells and HIV treatment failure

The pooled effects of CD4 cell count < 200 cells/mm3 (AOR = 7.2; 95% CI: 2.5–12.0), ≤ 100 cells/ mm3 (AOR = 2.1; 95% CI: 1.4–2.8) and < 50 cells/mm3 (AOR = 3.3; 95% CI: 1.4–5.3) as compared to those with > 200, > 100, and > 50 cells/mm3 on HIV treatment failure were estimated, respectively (Fig. 6).

**Fig. 7:**
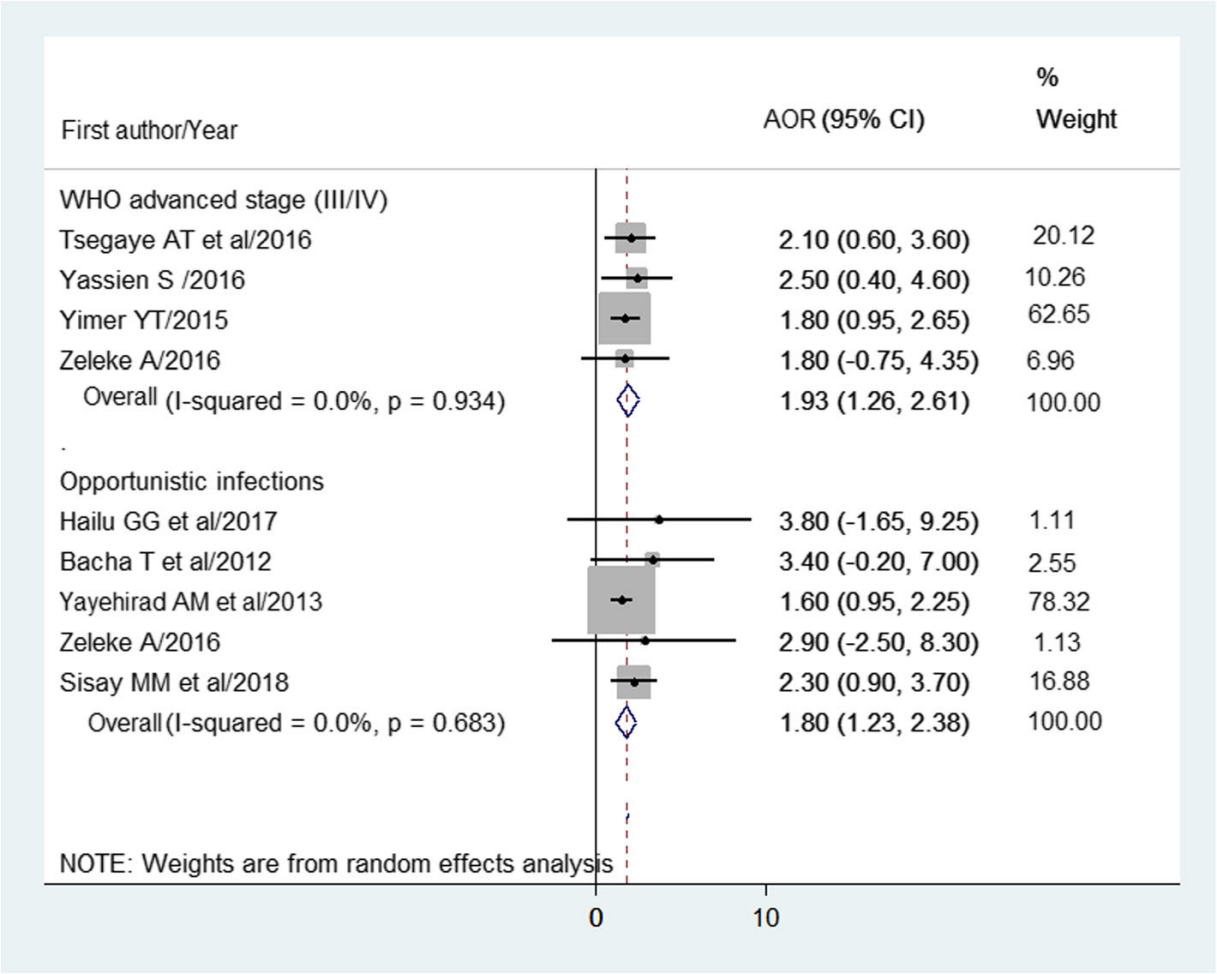
Forest plot of the adjusted odds ratios (AOR) with corresponding 95% CIs of studies on the association of WHO clinical stage, opportunistic infections, and HIV treatment failure

The pooled effect of being on WHO clinical stage III/IV found to be at higher risk (AOR = 1.9; 95% CI: 1.3–2.6) to HIV treatment failure as compared to stage II/I. The pooled effect of the presence of opportunistic infections (TB, diarrhea, pneumonia, other OIs) are more likely (AOR = 1.8; 95% CI: 1.2–2.4) to expose patients to HIV treatment failure (Fig. 7).

### Drug-related factors

Stavudine based regimen (AOR = 3.5; 95% CI: 1.3–10.6) [28], ART drug substitution (AHR = 1.7; 95% CI:1.1–2.7) [22], substitution of original regimen (AOR = 3.3; 95% CI = 1.6–6.7) [31], absence of PMTCT prophylaxis (AOR = 1.4; 95% CI: 1.2–2.5) [31], and using faith healing medicine (AOR = 8.1, 95% CI: 3.1–21.5) [30] were reported predictors of HIV treatment failure. Another study [30] showed that patients who didn’t have consultation were positively associated (AOR = 4.9,95% CI:1.5–15.8) with HIV treatment failure.

**Fig. 8:**
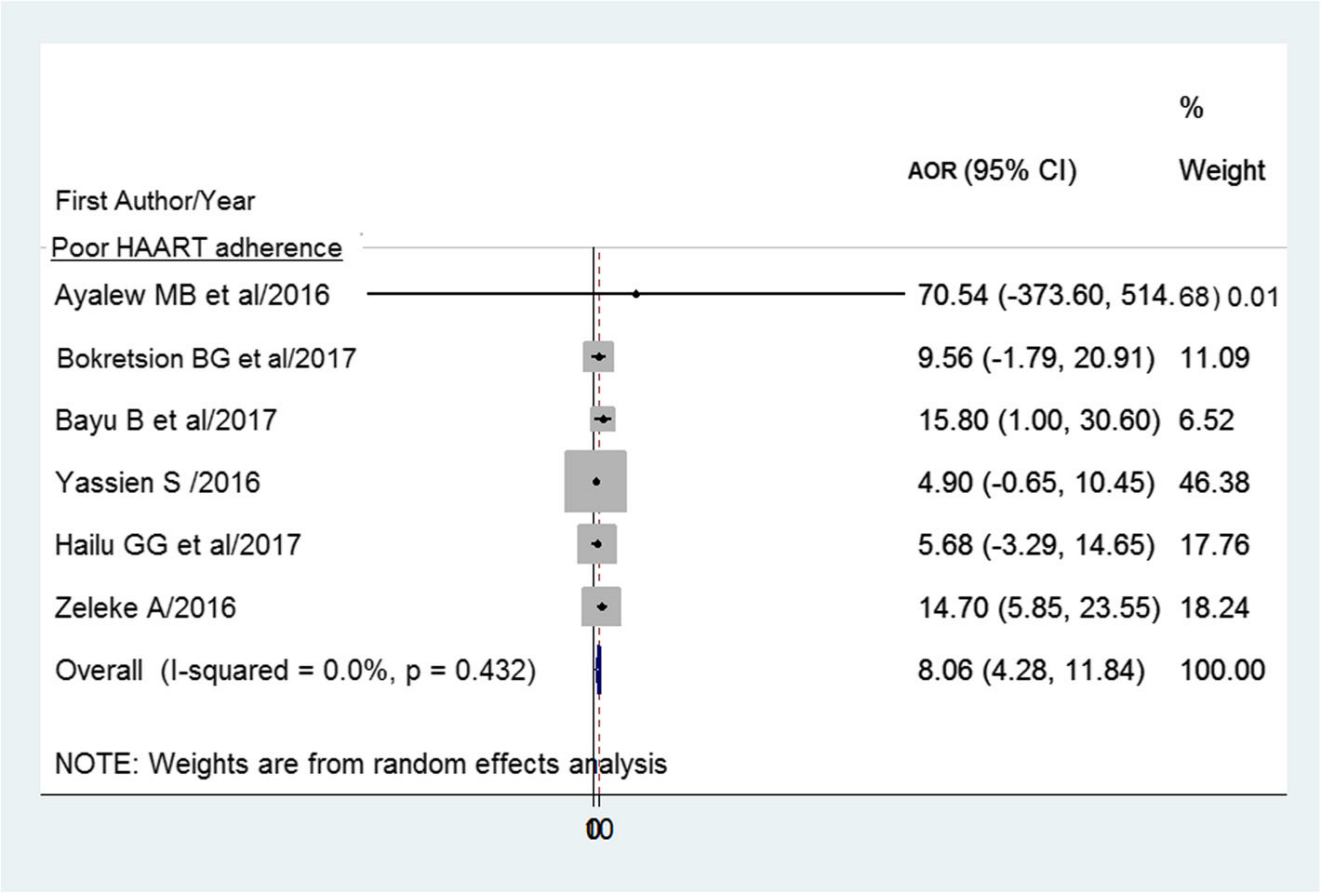
Forest plot of the adjusted odds ratios with corresponding 95% CIs of studies on the association of poor HAART adherence and HIV treatment failure

The pooled effect (AOR) of poor HAART adherence to HIV treatment failure was 8.1 (95% CI: 4.3–11.8) (Fig. 8).

## Discussion

Our study has two main findings related to the national prevalence and risk factors of HIV treatment failure. First, we noted that using the definition of HAART failure, HIV treatment failure was 15.9% (95% CI: 11.6–20.1%). This finding will support the global recommendation to switch patients on ART only after considering supplementary treatment failure prevention activities. In a resource-limited country, this finding could help to keep patients longer on first-line ART regimen which preserves the more toxic and expensive second-line ART regimen.

In Ethiopia, the threat of HIV treatment failure is becoming a continuing discussion. This might be due to poor HIV care services, delayed to recognize treatment failure, [34], late initiation of HAART [33], high burden of opportunistic infections [35], lack of appropriate nutritional support [36], ART-associated adverse reaction, [37] and frequent psychological problem [38, 39]. Besides, the absence of frequent therapeutic drug monitors and/ or resistance testing while the patient is still on the suspect or failing regimen. All four markers of lower socioeconomic status (financial hardship, non-employment, rented or unstable housing status, and non-university education) can be considered for the higher burden of HIV treatment failure in Ethiopia.

Though the WHO immunological criterion is a very low sensitivity and high specificity test [40], this finding showed that HIV treatment failure was higher (10.2%) when the immunological definition used than that of clinical (6.3%) and virological (5.6%) treatment failure. This variation might be due to studies included in the immunological definition of HIV treatment failure were more than studies used to pool the clinical and virological failure. Moreover, the lower prevalence of HIV treatment failure using the clinical definition might be due to limited diagnostic capabilities. It might be difficult to identify treatment failure in patients under clinical monitoring since not all HIV care clinic sites had a systematic approach and well-trained health professionals to collect data about opportunistic infections. Therefore, using viral load based HIV treatment failure could provide better prognostic information about the risk of developing active AIDS stage which will promote more effective second-line ART. However, in most Ethiopian health institutions, virological ART failure is likely to be under-diagnosed in the routine health system and might be limited to clinical and/or immunological failure as a result. Although only five studies were included to estimate virological ART failure, the third 90 target of UNAIDS seems to be achieved. There is a plan to achieve 90% of people who are receiving ART will have viral suppression by 2020 [8].

Based on the subgroup analysis, HIV treatment failure is lower in children. ART monitoring using clinical and immunological criteria is problematic in children, and misclassification rates using the WHO pediatric guidelines remain high [41].

This review found that lower CD4 cell count, being on the WHO clinical stage III/IV, presence of opportunistic infections, and poor HAART adherence were the predominant risk factors of HIV treatment failure.

It is estimated that lower CD4 cell count and advanced WHO clinical stage leads to HIV treatment failure. Other studies [42, 43] reported a similar finding in other settings. The presence of opportunistic infections, on the other hand, linked to CD4 cell level. As patients’ immune status becomes compromised, the rate of viral replication increases. CD4 cell count is the backbone of immunity construction that helps the human body to protect from the disease and can prevent HIV replication [44].

The presence of opportunistic infection (TB, diarrhea, pneumonia, other OIs) is more likely to exposed patients to develop HIV treatment failure. The patient gives more emphasis to the current problem than the chronic HIV, as such time interruption of taking a drug, lost follow-up, and other triple problems lead to HIV treatment failure.

Poor HAART adherence found to have a great impact on the occurrence of HIV treatment failure. It is widely agreed that once treatment is initiated, it should not be interrupted. In Ethiopia, within 07 days, nearly 11.3% of children have poorly adhered to ART [45]. It is expected that as duration increased the probability of ART interruptions would be more likely. The same in adult HIV patients, treatment interruption was falingl in the range between 11.8–25.8% [46, 47]. Acquired HIV drug resistance develops when HIV mutations emerge due to viral replication in individuals on imperfect ART adherence. Poor ART adherence could lead to incomplete viral suppression and causes HIV treatment failure. Global recommendations, like on-time pill pick-up, electronic or paper-based appointment scheduling, SMS or telephone call reminders, peer counseling, cognitive behavioral therapy, and reduction of the HIV-associated stigma that prevent missing of ART drugs are not well implemented in Ethiopia.

## Conclusions

HIV treatment failure in Ethiopia found to be high. Being on advanced WHO clinical stage, presence of opportunistic infections, and poor adherence to highly active antiretroviral therapy were the contributing factors of HIV treatment failure. The current finding will have health policy and clinical implications for therapeutic management decisions. Early identification of ART treatment failure allows patients to have a higher chance of success when switching to a second-line ART. A report on HIV treatment failure will be used to monitor the progress of the national action plan of 90–90-90 strategies.

## Data Availability

All data generated or analyzed during this study are included in this published article and its supplementary information files.

## Abbreviations

AIDS: Acquired Immunodeficiency Syndrome
AOR: Adjusted Odds Ratio
CI: Confidence Interval
HAART: Highly Active Antiretroviral
HIV: Human Immunodeficiency Virus
WHO: World Health Organization
